# High-Sensitivity Troponin: A Review on Characteristics, Assessment, and Clinical Implications

**DOI:** 10.1155/2022/9713326

**Published:** 2022-03-28

**Authors:** Diana Raluca Lazar, Florin-Leontin Lazar, Calin Homorodean, Calin Cainap, Monica Focsan, Simona Cainap, Dan Mircea Olinic

**Affiliations:** ^1^Emergency County Hospital for Children, Pediatric Clinic No. 2, Department of Pediatric Cardiology, Cluj-Napoca, Romania; ^2^“Iuliu Hatieganu” University of Medicine and Pharmacy, Department No. 11, Oncology, Cluj-Napoca, Romania; ^3^County Emergency Hospital Cluj-Napoca, Medical Clinic No. 1, Interventional Cardiology Department, Romania; ^4^“Iuliu Hatieganu” University of Medicine and Pharmacy, Cardiology Discipline, Cluj-Napoca, Romania; ^5^“Prof. Dr. Ion Chiricuta” Oncology Institute, Cluj-Napoca, Romania; ^6^Nanobiophotonics and Laser Microspectroscopy Center, Interdisciplinary Research Institute on Bio-Nano-Sciences, Babes-Bolyai University, Cluj-Napoca, Romania; ^7^“Iuliu Hatieganu” University of Medicine and Pharmacy, Department No. 9, Mother & Child, 400012 Cluj-Napoca, Romania

## Abstract

The use of high-sensitivity cardiac troponin (hs-cTn) assays has become part of the daily practice in most of the laboratories worldwide in the initial evaluation of the typical chest pain. Due to their early surge, the use of hs-cTn may reduce the time needed to recognise myocardial infarctions (MI), which is vital for the patients presenting in the emergency departments for chest pain. The latest European Society of Cardiology Guidelines did not only recognise their central role in the diagnosis algorithm but also recommended their use for rapid rule-in/rule-out of MI. High-sensitivity cardiac troponins are also powerful prognostic markers for long-term events and mortality, not only in a wide spectrum of other cardiovascular diseases (CVD) but also in several non-CVD pathologies. Moreover, these biomarkers became a powerful tool in special populations, such as paediatric patients and, most recently, COVID-19 patients. Although highly investigated, the assessment and interpretation of the hs-cTn changes are still challenging in the patients with basal elevation such as CKD or critically ill patients. Moreover, there are still various analytical characteristics not completely understood, such as circadian or sex variability, with major clinical implications. In this context, the present review focuses on summarizing the most recent research in the current use of hs-cTn, with a main consideration for its role in the diagnosis of MI but also its prognostic value. We have also carefully selected the most important studies regarding the challenges faced by clinicians from different specialties in the correct interpretation of this biomarker. Moreover, future perspectives have been proposed and analysed, as more research and cross-disciplinary collaboration are necessary to improve their performance.

## 1. Introduction

As ischaemic heart disease continues to be a leading cause of death worldwide, a fast and accurate diagnosis is critical in patients with presumed acute coronary syndrome (ACS). In order to achieve this goal, not only a precise definition of the myocardial infarction (MI) but also a straightforward diagnosis algorithm was needed. In the last decades, a new cardiac biomarker, the high-sensitivity troponin, has emerged as an indispensable tool, as it has increased diagnostic accuracy in patients with acute chest pain in comparison with conventional cardiac biomarkers [1].

While the first definition of MI was mainly based on the electrocardiographic (ECG) findings, after multiple reviews, the European Society of Cardiology published in 2018 the 4th universal definition of MI, in which a biomarker, the high-sensitivity cardiac troponin (hs-cTn), acquired a central role in the diagnosis algorithm. According to this definition, in order to establish the diagnosis of MI, myocardial injury defined by an elevated cardiac troponin value is mandatory, alongside additional criteria [2]. Therefore, the ability of hs-cTn to detect very small infarcts that otherwise would have not been considered MI underlines the important clinical implications of this biomarker and the need of its correct assessment.

The aim of this paper is to present the most important characteristics of this biomarker, including the assessment methods, clinical implications, and possible future perspectives, in order to improve its use in daily practice.

## 2. Brief History

The first biomarker used for the diagnosis of AMI was aspartate transaminase (ASAT), first described by Karmen et al. [3] and later incorporated in the WHO definition of AMI in the 1960s. As this enzyme is not cardiospecific, several other enzymes, such as lactate dehydrogenase, creatine kinase (CK), myoglobin, and CK isoenzyme MB (CK-MB), were later described and used in the diagnosis algorithm, with increased sensitivity and specificity, thus improving the diagnosis accuracy. Even if the overall performance of the new enzymes was acceptable, with little differences among them, their low sensitivity in the first 4-6 hours, a time frame crucial for the early diagnosis and patients' prognosis, required the search for a new biomarker.

**Figure 1:**
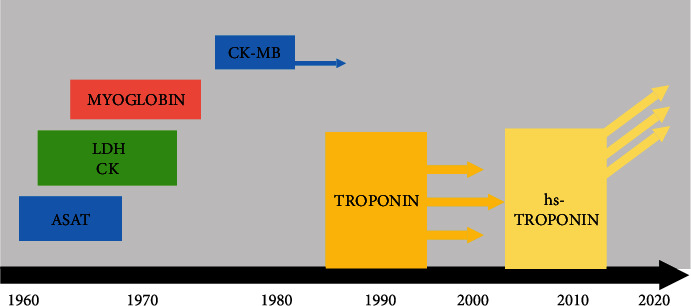
Brief history of the cardiac biomarkers. ASAT = aspartate transaminase; LDH = lactate dehydrogenase; CK = creatine kinase; CK-MB = creatine kinase isoenzyme MB.

**Figure 2:**
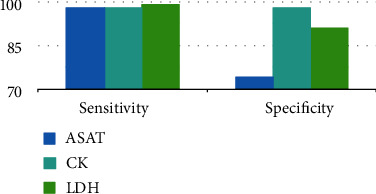
Sensitivity and sensibility of ASAT, CK, and LDH. ASAT = aspartate transaminase; LDH = lactate dehydrogenase; CK = creatine kinase.

**Figure 3:**
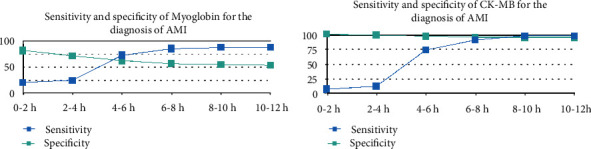
Sensitivity and sensibility over several periods of time from the onset of AMI. AMI = acute myocardial infarction; CK-MB = creatine kinase isoenzyme MB.

In the 1990s, a new era arose in the biochemical diagnosis of AMI, as a sensitive and reliable radioimmunoassay was developed to detect serum troponin, which was first described in 1965. Cardiac troponins T and I appear in the serum early after the onset of AMI, reaching a peak after 12-48 h and remaining elevated for 4-10 days, with a sensibility close to 100% in detecting AMI at 6-12 h after acute chest pain onset [4]. As this new biomarker revolutionized the diagnosis algorithm of AMI, improving its assessment became the major focus in the next years. After the high-sensitivity assay started to be used in 2007-2010, hs-cTn became the gold standard for laboratory tests, moreover playing a central role in the diagnosis algorithm in the last universal definition of MI. The evolution of the biomarkers used for the diagnosis of AMI is represented in Figure 1 and the main differences in their performance may be seen in Figures 2 and 3 and Table 1 [5–7].

## 3. Cardiac Troponins: From Promising to Gold Standard

### 3.1. What Is the Cardiac Troponin***?***

Troponins are structural proteins found in the troponin complex within skeletal and cardiac muscle thin filaments. The troponin complex consists of three subunits (I, T, and C) and along with calcium ions plays an important role in the regulation of muscle contraction [8]. Each molecule has a specific role in the muscle contraction process: troponin T attaches the troponin complex to the actin filament, troponin C acts as the calcium binding site, and troponin I inhibits interaction with myosin heads in the absence of sufficient calcium ions [4]. During the depolarization of the cardiac myocyte, calcium enters the cell through voltage-gated L-type Ca2+ channels (LTCCs), which are brought close to the ryanodine receptor type-2 (RyR2) channels through a T-tubular network of membrane invaginations. This process determines a high Ca2+ release from the sarcoplasmic reticulum (SR) to the cytosolic space, promoting Ca2+ binding to cTnC, and induces structural changes in the cTn complex. The resulting shift of tropomyosin away from the active site of actin allows the myosin heads of the thick filament to interact with the now-exposed myosin-binding site of the actin filaments, thereby producing contraction of the sarcomere and, consequently, the myocardium [9].

While troponin C is synthesised in both skeletal and cardiac muscles, troponin T and I are mainly localised in the myocardium, thus being referred to as cardiac troponin (cTnI and cTnT). Even if there are several contradictory studies indicating their presence in other sites, such as tunica media of the vena cava and pulmonary veins, aorta, trachea, gut, urinary bladder, or even the skeletal muscle, it is generally accepted that these biomarkers possess the greatest specificity in identifying myocardial injury [10].

The largest amount of troponin is found as part of the troponin complex in the cardiac sarcomere and only about 5% is free in the cytoplasm. This distribution determines, in the case of a myocardial injury, an initial rapid release from cytoplasm, with a further gradual release of structural origin complexes [8]. This biphasic release pattern is of great importance for the diagnosis of myocardial infarction, as the initial appearance of troponin in the serum has been also noticed in nonnecrotic myocyte ischemia, whereas continuous cTn release is highly suggestive for necrosis, as the real half-time of troponin is of approximately 2 hours.

This particular pharmacokinetics has been explained by multiple experimental studies which demonstrated that ischemia induces bleb formation on the surface of cardiac myocytes. Blebs are bubbles in the plasma membrane, which are formed and grow as a response to ischemia. In case of prolonged ischemia, the blebs rupture and this is followed by prolonged troponin release, as in the case of myocardial infarction. On the other hand, in transitory ischemia, the blebs are either resorbed or shed in the circulation, in this last case releasing cytoplasmic contents, including free troponin [11].

Nevertheless, it has also been demonstrated that very low concentrations of cTn may be found in the blood of healthy individuals due to the cardiomyocyte turnover. Bergmann O et al. analysed the ^14^C concentration from the DNA extracted from the cardiomyocyte nuclei and reported a cardiomyocyte renewal at a decreasing rate from 1% annually at the age of 20 to 0.3% at the age of 75, for about 40% of the total cardiomyocytes [12]. Besides bleb formation and possible physiological cardiac cell turnover, there are several other mechanisms involved in the troponin release, such as pathological increased cell membrane permeability, myocyte apoptosis, integrilin-stimulated mechanical stretch of cardiomyocytes, or proteolytic degradation [13, 14]. In isolated rat hearts, Feng et al. increased the end-diastolic pressure of the left ventricle and found that the elevated preload, which can also be seen in heart failure, is sufficient to determine TnI degradation independently of ischemia. This process may be explained by the preload-induced mechanical strains, leading to *μ*-calpain activation and subsequent TnI proteolysis and circulation release of the fragments in the absence of sarcolemmal disruption [14]. On the other hand, mechanical stretch of cardiomyocytes is capable of initiating a cascade of intracellular reactions, such as increased calcium and nitric oxide concentrations, as well as the activation of several proteases, which subsequently are able to degrade cTnI intracellularly, leading to the release of cTnI and its degradation products into the circulation [13].

All of these pharmacokinetic properties imply a delay in the identification of the elevation cause, which may be detrimental in the early stages of a myocardial infarction. However if there is high clinical suspicion of myocardial ischemia, a significant elevation of cardiac troponin is highly indicative of MI.

### 3.2. Detection Methods and Analytical Characteristics

As the troponins are one of the main pillars in AMI diagnosis, the analytical performance of the detection method used is of crucial importance. The most frequently encountered methods nowadays are immunochemical methods, such as enzyme linked immunoassay (ELISA), immunofluorescence assay, radioimmunoassay (RIA), and immunochemiluminescence assay.

The basis of these assays are similar: there is an immunological phase, in which there is a specific antibody (anti-cTn)—antigen (cTn N-terminal amino acid) interaction, a second phase in which there is a either an enzymatic reaction or another antibody-antigen reaction (depending on the method used), followed by a final detection phase, which varies according to the detection method being used: for ELISA a spectrophotometer is being used to assess colour intensity, for RIA a radiometer is used to detect radionuclides emissions and for immunofluorescence, fluorophores are detected using a fluorometer. Signal strength is directly proportional to the number of troponin molecules detected, which makes quantification possible. Several studies have compared the analytical performance of different methods. For example, in a recent study, Sörensen et al. assessed the prognostic value of hs-cTnI in patients with suspected AMI analyzed by a novel (Singulex Clarity cTnI) and established hs-TnI assay (ARCHITECT STAT hs-TnI, Abbott), which is a two-step immunoassay using chemiluminescent microparticle immunoassay (CMIA) technology for the quantitative determination of cardiac troponin-I. The authors reported no differences between the two assays in predicting adverse events, with several other studies reporting similar results, but underlying the urgent need for standardisation when measuring hs-cTn [15].

Due to the important cross-reactivity of diagnostic antibodies and low sensitivity of the first generation methods, the second-generation methods were developed. This was the moment when cTnT eventually surpassed all the conventional existing biomarkers in the ability of early diagnosis of AMI. Having a higher sensitivity and specificity, the determination of cTnT using this new assay became a recommendation for routine practice in a joint document of European and American Cardiology Societies in 2000 [16]. After continuously improving the determination method, resulting in almost no cross-reactivity and improved analytical characteristics, nowadays the “fifth generation” immunoassays represent the gold standard in the determination of cTnT. The method has a detection limit of 1-10 ng/L with 20-30 minutes required for testing [17]. Regarding the cTnI, the evolution of the immunoassays has been similar to the cTnT and more than 30 commercially tests are now available for their determination.

Although the diversity of methods led to a constant improvement in the assessment of hs-cTn, the lack of standardization is a major disadvantage as the results obtained with different kits may present significant differences, mainly due to the fact that different anti-hs-TnI antibodies directed to different antigenic determinants of the cTnI molecule are used in different kits; thus, the standardization of immunochemical methods represent the next border to be surpassed [18].

High-sensitivity troponin assays are used to detect troponins, but at a much lower concentration than classical assays. These assays offer several advantages, first of all being the fact that they are highly sensitive, thus providing faster recognition of AMI (rule-in/rule-out). They are also very precise, with small coefficient of variation even at the 99th percentile, in the reference population [19].

What is more, since hs-cTn assays are being used, a lot of insight in troponin biology has been uncovered. It has been shown that there is a variability of troponin levels even in healthy patients, men presenting a higher level than the women, a circadian variability, with their values being slightly higher in the morning in healthy patients, age related variability. It has also shown differences in variability between healthy patients and patients with comorbidities, such as CKD and diabetes. This circadian variability is noteworthy, considering the fact that an increase of troponin levels in the early morning hours could be erroneously interpreted as myocardial damage.

The currently used 5th generation immunoassays distinguish from the previous generations methods by the improved analytical characteristics, which are mainly represented by (1) limit of blank, (2) limit of detection (LoD), (3) the limit of quantitation (LoQ) or functional sensitivity, (4) 99th percentile (general), (5) 99th percentile (taking into account sex characteristics), (6) percentage of measurable values in healthy individuals, (7) coefficient of variation (CV%), and (8) 99th percentile/LoD ratio [20]. Even if these main characteristics may differ from a manufacturer to another, in an expert consensus, Apple et al. stated that a reliable assay should have a coefficient of variance of <10% and concentrations below the 99th percentile should be detectable above the assay's limit of detection for >50% of healthy individuals in the population of interest [21]. The analytical characteristics, which are defined and described in Table 2, should be taken into consideration not only by manufacturers and researchers but also by the practitioners, as they influence the clinical use of this valuable laboratory test.

### 3.3. Clinical Implications

#### 3.3.1. Role of hs-cTn in the Diagnosis of Cardiovascular Pathologies

The most important clinical implication emerged from the development of high-sensitivity assays for cTn is the central role that this biomarker gained in the diagnosis of myocardial infarction. In “Fourth universal definition of myocardial infarction”, cTnI and cTnT are recommended to both rule in and rule out myocardial injury and thus to define every subtype of MI. To establish the diagnosis of an acute MI, a rise and/or fall in cTn values with at least one value above the 99th percentile upper reference limit (URL) is required, coupled with a high clinical and/or ECG likelihood of myocardial ischaemia [2].

Moreover, the major advantages that these assays have compared to standard assays are a higher negative predictive value for AMI and a significant shorter “troponin-blind” period, which led to the development of more rapid ‘rule-in' and ‘rule-out' MI algorithms, translating into shorter stays in the emergency department and lower costs. According to the latest ESC Guideline on the management of acute coronary syndromes in patients presenting without persistent ST-segment elevation, the best option is the use of a 0/1 hour algorithm or 0/2 hours, which result in a significant increase in the detection of type 1 and 2 MI, although relevant 1 h changes are assay dependent. Optimal thresholds for rule-out were selected to allow for a minimal sensitivity and NPV of 99%. Optimal thresholds for rule-in were selected to allow for a minimal positive predictive value (PPV) of 70% [22].

Although the safety and efficacy of these algorithms have been proven in several large studies [23], they have to be implemented taking into account multiple factors that can influence the interpretation (age, renal dysfunction, sex, and time from chest pain onset) and should only be used in conjunction with detailed assessment of chest pain characteristics and ECG. Even if a MI is ruled-out, elective noninvasive or invasive tests can be indicated, in respect to the clinical likelihood, except for the cases where a clear alternative diagnosis is found, which do not require supplementary tests. However, in patients who do not qualify for ‘rule-out' or ‘rule-in', a third measurement of cardiac troponin at 3 h and echocardiography is required, while the third measurement is mandatory in the case of recurrent chest pain or high-risk patients.

Although the high-sensitivity assays reduced the time needed to recognise a MI, there are still many patients that rule in after 6 hours, or even more than 12 hours, as they present late after the onset of acute MI and because they are on the downslope of the time-concentration curve may require longer periods of time for a changing pattern to be detected [24].

Another important clinical implication in the interpretation of hs-cTn is the difficulty sometimes encountered in distinguishing acute myocardial injury from chronic conditions associated with a chronic elevated basal level of troponins. Even if many times difficult, the demonstration of a rising and/or falling pattern is needed to distinguish between the 2 settings, while the absence of dynamic changes in nonsuggestive clinical presentations should orientate the practitioner into searching for alternative causes for troponin raise.

The hs-cTn are also important in non-ACS, as low levels of troponin can be detected in many patients with stable angina and increased troponin levels are associated with adverse outcome in these patients, but as large trials are still needed to verify the utility of systematic assessment, routine use of biomarkers or other imaging tests for CAD are not recommended in asymptomatic patients [25].

#### 3.3.2. hs-cTn as a Prognostic Factor

The hs-cTn assessment also plays an important role as a prognostic factor in AMI but also in chronic coronary syndromes and general population. While hs-cTn T and I share comparable accuracy in the AMI diagnosis, Haaf et al. showed higher accuracy for cTnT in predicting long-term mortality, although further dynamic measurements do not improve the initial risk stratification [26]. In this trial, hs-cTnT outperformed hs-cTnI in its prognostic accuracy both in all patients and in important subgroups, including acute MI at presentation, preexisting CAD, impaired renal function, or patients older than 70 years. In another study, Welsh et al. analysed the Tn levels in the general population and concluded after a median of 7.8 years that elevations in cTnI are more strongly associated with some CVD outcomes, whereas cTnT is more strongly associated with the risk of non-CVD death, while both cTn are associated with heart failure and cardiovascular disease death [27]. The authors also reported similar association with ischemic stroke for both troponins and proposed some theories to explain the unexpected differences found between the two troponins, including the transiently expression in fetal skeletal muscle of cTn-T, as well as the different upstream genetic determinants (some of the genes associated with cTnT are highly abundant in cardiac z-disc structures, while for cTnI, the most important genes are part of the kallikrein-kinin axis and loci have been associated with vasoactive peptides).

Even though in the RUTI-STEMI study new-generation troponins levels were not shown to predict clinical events in STEMI patients [19], there is increasingly important data demonstrating not only increased mortality for patients presenting with elevated troponin levels at admission but also a linear correlation between the troponin levels and worse outcomes [28], with an increased power of further risk stratification when combined with electrocardiography and the CK-MB level [29]. Moreover, a large meta-analysis including more than 150.000 patients from 28 relevant prospective studies reported an independent association between high cardiac troponin concentration within the normal range and CVD risk, both for stroke and coronary disease [30]. All of these studies may suggest that the prognostic value of hs-cTn could also help to guide therapy, but as the data is still lacking, large studies are needed to demonstrate if specific thresholds of high-sensitivity assays might also be used to guide therapeutic strategies.

Besides ACS, there are several other clinical settings in which the hs-cTn has been demonstrated to be a reliable predictive biomarker. In acute pulmonary embolism, even though 60% of the patients present with elevated hs-cTn levels, the detection of an elevated level of cTnI or T assessed by high-sensitivity assays are associated with an increased risk of mortality both in unselected patients and haemodynamically stable ones. Even if in normotensive patients the specificity and PPV of this biomarker are low, but with a high NPV, when interpreted in combination with clinical and imaging findings, they may improve the identification of an elevated PE-related risk [31, 32]. Elevated hs-cTn levels are also a good prognostic marker in patients with congenital heart disease [33], infective endocarditis [34], acute pneumonia [35], atrial fibrillation (AF) [36], intracranial haemorrhage [37], and heart failure (HF) [38]. For example, in a study including 171 patients with acute pneumonia aged >75 years old, the elevation of cardiac troponin I was strongly associated with short-term mortality and a higher risk of in-hospital complications, suggesting that troponin measurement at admission for patients with acute pneumonia could help clinicians to individualise the CV prevention strategies in order to improve the short and long-term outcomes [35].

An important work worth to mention is the study conducted by Hijaz et al. who developed and validated a new biomarker-based risk score for predicting stroke in AF in a large number of patients with AF treated with oral anticoagulants. The ABC-stroke score (age, biomarkers, and clinical history) included the cardiac biomarkers N-terminal fragment B-type natriuretic peptide (NT-proBNP) and hs-cTn and was reported to predict stroke or systemic embolism with a significantly higher accuracy than the currently used CHA2-DS2 -VASc score. Although the mechanisms by which these biomarkers significantly improve the risk prediction in patients with AF, this study is suggesting that the use of the ABC score could improve the outcome of AF patients [36]. Even though the precise mechanism of cardiac troponin elevation in patients with intracranial haemorrhage (ICH) is still unknown, several hypotheses have been proposed. As excessive catecholamine release has been shown to induce myocardial injury, it may be assumed that the catecholamine surge found in these patients is responsible for the troponin elevation. Moreover, the systemic inflammatory responses activated by the ICH might also play an important role in the myocyte injury and cell death processes [37]. In patients with heart failure, the hs-cTn provide important prognostic information on the future risk of HF's manifestation in asymptomatic subjects and the risk of fatal events and primary/readmissions in the hospital in those with already established symptomatic acute, acutely decompensated and chronic stable HF [38].

Adjusted to age, even if asymptomatic old patients with left ventricular hypertrophy and elevated hs-cTn are more likely to develop heart failure, this biomarker is not a useful tool to detect the HF phenotype, as hypothesised in several studies [39, 40].

However useful in the clinical setting of an AMI, an elevated troponin is not always a precise indicator of the underlying disease, which is why the above mentioned diagnosis is considered only by incorporating the clinical, electrocardiographic and echocardiographic data. We can safely state that troponins are a very specific marker for myocardial damage, but do not have sufficient specificity for AMI, as they increase in a number of conditions leading to myocardial cell damage, or affecting myocardial tissue. Nevertheless, it should be reminded that false-positive results can be noted as a consequence to cross-reactions of diagnostic anti-cTnI and anti-cTnT antibodies with skeletal troponins, the influence of heterophilic antibodies, alkaline phosphatase, biotin, hemolysis, icterus presence, and lipemia [10].

Taking all these into consideration, the practitioner should always keep in mind that an elevated level of hs-cTn is not always pointing to an ACS, and in particular cases, not even myocardial injury.  Table 3 lists the main causes for elevated cardiac troponins [2, 9, 10].

### 3.4. Sex Differences

Multiple relevant studies found a higher incidence of type 2 MI, spontaneous coronary artery dissections, Takotsubo syndrome, and MINOCA in women but also showed worse outcomes for CAD, as they usually present with atypical symptoms and require a longer time until the correct diagnosis is established.

Regarding these sex-specific differences, there is currently sufficient data which demonstrated that hs-cTn have significantly lower values among women compared with men [2]. One of the explanations for higher levels of cTn in men could be the larger left ventricular mass, for which Gore et al. proposed sex-specific 99th percentile URLs for hs-cTn assays, in order to avoid overdiagnosis of MI in men [41]. After other several studies validated these findings, the currently used guidelines recommend the use of sex-specific 99th percentile URLs for hs-cTn assays [2]. Regarding the prognostic value of troponins in women, Egger et al. concluded that despite lower values, cTnI above the tested 99th percentiles exhibits stronger prognostic information in women with NSTE-ACS compared with men [42]. Moreover, the authors reported potentially improved diagnostic and prognostic information for sex-specific cut-off values of hs-cTn. Several other mechanisms have been proposed in order to explain the sex-specific differences besides the body composition and left ventricular mass, including different rates of cardiomyocyte apoptosis due to cardiac remodeling, unequal myocardial response to ischemia and reperfusion, variable grade of coronary atherosclerosis and the presence of collateral blood flow or protective role of estrogens due to their antioxidant properties [43]. However, some large studies failed to demonstrate improved diagnostic accuracy for sex-specific thresholds [44].

Moreover, the reclassification from unstable angina to non-ST elevation MI did not seem to impact short-term or long-term prognosis in large cohorts, although the use of sex-specific cut-offs for hs-cTn resulted in increased rates of acute MI in women and decreased rates for men [43]. Taking into consideration the high heterogeneity between the results of these studies, the need for further research in this area is still obvious.

### 3.5. High-Sensitivity Cardiac Troponin and Kidney Disease

Chronic kidney disease is associated with worse outcomes in coronary artery disease, end stage renal disease patients being at high risk for adverse cardiovascular events [45]. The management of these patients represents one of the most challenging tasks in the setting of a MI, due to atypical presenting symptomatology, higher risk of post-intervention complications and increased bleeding and ischaemic risk [46]. Moreover, even the diagnosis of coronary disease in these patients represents a challenge, as the majority of patients have elevated hs-cTn values at baseline with a high prevalence which cannot be explained only by the reduced renal clearance [47]. Even though the aetiology for the elevation is not completely understood, several mechanisms have been proposed, the most popular being (1) long-term exposure to uremic toxins and/or comorbidities, leading to myocyte injury and (2) decreased renal clearance. Moreover, a study conducted by Chesnaye et al. demonstrated a negative relationship between hs-cTnT level and renal function over time, with lower CKD stage being independently associated with a steeper hs-cTnT increase over time [48]. What is more, Kavsak et al. showed that both hs-CTnI and hs-CTnT values correlate negatively with eGFR, concluding that for an accurate risk stratification in the diagnosis process of an AMI, in patients with CKD, eGFR must be known and taken into account [49]. In a small cohort study, Ünlü et al. described a significant increase in hs-TnT induced by hemodialysis, thus warning on the risk of unnecessary interventions in these patients, when the management is mainly based on laboratory findings [50].

Based on these observations, the question whether specific cut-offs should be used for these patients emerged. For example, Twerenbold et al. suggested that hs-Tn cut-off levels for patients with CKD should be up to 3.4-fold higher than in patients with no renal pathology [51], but larger studies suggest that there are no differences in the effectiveness of serial changes of the cTn levels in diagnosing MI between general population and CKD patients [52]. Thus, the current ESC Guidelines suggest that a rising and/or falling pattern should remain the main method in detecting ACS and do not recommend different criteria for the cTn decision levels in CKD, although additional imaging studies may be necessary to determine the appropriate diagnosis [2].

Even though three observational studies that evaluated the diagnostic performance of hs-cTn by comparing the performance of the 0/1-hour ESC algorithms of rapid rule-in and rule-out in patients with and without renal dysfunction using both hs-TnT and hs-TnI showed lower specificity in CKD patients, the sensitivity of the test remained close to 100%, hs-cTnI being able to identify over 90% of MI within 3 hours [46].

Moreover, as the risk of cardiac events at 1 year is two-fold greater in patients with troponin concentrations > 99th percentile, independent of the diagnosis [53], the prognostic role of this biomarker remains to be of incontestable value. The TROPIC study demonstrated the prognostic value of both T and I hs-Tn for short-term all-cause death, but not all long-term prognostic, with TnI being more accurate than hs-TnT in detecting coronary disease [54].

Taking all these into consideration, as renal dysfunction only reduces the rule-in but not also the rule out performance of the hs-cTn in MI and higher mortality rates are associated with elevated levels of hs-cTnI, this biomarker continues to play the central role in the ACS diagnosis algorithm even for patients with CKD [55], but future research is still open in better understanding its optimal interpretation in this setting.

### 3.6. Troponin in Young Patients and Children

Another sensitive topic in the clinical interpretation of the hs-cTn assessment is represented by children and young patients, as MI is relatively uncommon among young subjects, with an incidence of 2-10%, and thus, it cannot explain the troponin elevation in most of the patients. Several studies found arrhythmia, myocarditis and vasospasm secondary to drug use to be the most common cardiac causes for troponin elevation in younger than 20 years old patients presenting with chest pain, but keeping in mind that noncardiac causes such as central nervous system pathology, end-stage renal disease, chest wall trauma, pulmonary embolism, and rhabdomyolysis/myositis are more common in these patients [56, 57]. In a study including 212 patients < 22 years old presenting in the emergency department with chest pain, only 17% had a significant raise in the hs-cTn levels, from which only one had an AMI and the authors concluded that due to the low likelihood for an ACS to be the aetiology of chest pain, there is no need for an immediate transfer to an adult facility for these patients [58].

However, the prognostic role of the troponins is of great importance in the young population, as a large study conducted by Wu C demonstrated that young patients in which elevated troponins were detected have a significantly higher all-cause mortality and worse long-term outcomes when compared with those who present with myocardial infarction, regardless of the aetiology [56].

Regarding paediatric patients, supraventricular tachycardias represent an important cardiac cause for ED presentation, but in this setting, hs-cTn possesses a low predictive value for the outcomes [59]. On the other hand, as the incidence of neoplastic pathology is increasing in children, chemotherapy-induced cardiotoxicity became an important health problem. In this setting, both I and T cardiac troponins were shown to have an important prognostic role, being able to predict up to 3 months ahead the development of a decrease in LVEF, but there is still important controversy regarding this topic. As several studies demonstrated the role of elevations of high-sensitive cardiac troponin-I in predicting subclinical cardiomyocyte damage, other small studies proved a very low sensitivity for subclinical heart disease when troponin T was used [60, 61]. What is more, as hs-cTnI levels can be elevated in up to 13.1% of healthy children, many studies suggest the need of using adjusted cuts-off values for this patients [62]. Using a proper cut-off value, this biomarker could play a major role in optimizing cardioprotective treatments in patients receiving anthracycline therapy [63], but further large studies are needed.

Nevertheless, there are several other studies demonstrating the predictive value of hs-cTn assays for a large spectrum of pathologies resulting in myocardial injury. Li et al. demonstrated that the assessment of hs-cTnI using specific cut-offs can predict myocardial injury caused by neonatal asphyxia at an early stage, with a higher diagnostic precision that other conventional biomarkers such as CK-MB, BNP, and myoglobin, with similar results being obtained for hs-cTnT in another study [64, 65]. Another interesting use of the hs-cTn assessment is the evaluation of HIV-exposed uninfected children (HEU), as Wilkinson et al. found a significant association between elevated cTnT and higher mean left-ventricular end-diastolic posterior wall thickness, suggesting that cardiac biomarkers may help identify HEU children who require further cardiac evaluation including echocardiography [66]. In another study, Das et al. found higher levels of hs-cTnT in children with acyanotic congenital heart disease, suggesting that the assessment of this biomarker could become an important tool in stratifying children for early surgical intervention, before the occurrence of irreversible myocardial damage [67].

In summary, while in adult patients the role of hs-cTn is very well documented in different clinical scenarios, with little gaps in evidence, data is still scarce about the role of this biomarker in young adults and children, but as more studies are demonstrating potential useful benefits from its use in several settings, further studies are needed to validate the results.

### 3.7. Troponins in the COVID-19 Era

The year 2019 marked the beginning of a new era, as the COVID-19 pandemic broke out, imposing devastating effects on populations, social structures, and economic growth, with researchers engaged in a desperate continuous work in finding not only effective diagnostic and prognostic tools but also treatment and prevention strategies. The natural evolution of this disease is very variable, from completely asymptomatic to “severe disease.” As patients with “severe disease” can often deteriorate in a fast manner, it is very important to use proper prognostic tools, in order to efficiently triage patients, personalize treatment and monitor clinical progress.

An important number of studies found significant association between the presence of several comorbidities and the risk of intubation and death, but certain biomarkers such as elevated D-dimer levels, C-reactive protein (CRP), LDH, and high-sensitivity cardiac troponin also proved to be reliable in predicting worse outcomes [68].

One of the early findings in the COVID-19 patients was the association between the cardiac damage, which was more common in the “severe disease” patients and poor outcomes [69]. Several mechanisms have been proposed to explain the high occurrence of myocardial injury in COVID-19 patients, including direct damage to the cardiomyocytes, systemic inflammation, myocardial interstitial fibrosis, systemic interferon-mediated immune response, exaggerated cytokine response by type 1 and 2 helper T cells, coronary plaque destabilization, oxygen supply-demand mismatch, microembolic infarcts, hyperadrenergic state, and pulmonary embolism [70]. Thus, the early assessment of elevated hs-cTn not only identifies the patients with severe disease but also is associated with a 71% increase in in-hospital mortality and a 2-fold increase in the risk of major complications, including sepsis, acute kidney failure, multiorgan failure, pulmonary embolism, and major bleeding [71], with some studies suggesting a higher predictability for poor prognostic than underlying cardiovascular disease alone [68].

Moreover, as the incidence of elevated hs-cTn varies between 20 and 45% at admission in the COVID-19 patients and several studies demonstrated that even milder elevations are associated with poorer outcomes, serial assessments may improve prognostic stratification. In a study conducted by Zaninotto et al., many patients presented several peaks of value, which were preceded by severe hypoxia, suggesting recurrent type 2 AMI in several different moments during hospitalisation. These observations are in favour of serial hs-cTn monitoring, as this strategy can offer additional data regarding the type and the severity of myocardial injury associated to COVID-19 disease; thus, practitioners can restratify the risk and prognosis and also can make changes to the management strategy [72].

With a still on-going pandemic, more studies will be conducted in order to better understand the role of troponins in this setting, and as multiple risk scores are already being tested, hs-cTn will remain a central piece for predicting the outcomes in COVID-19 patients and ultimately in the decision-making process.

## 4. Potential Challenges and Future Perspectives

Even though tremendous progress has been achieved in the understanding of the hs-cTn and this biomarker has changed many daily-practice strategies, there are still many challenges to be overcome regarding not only its assessment but also its possible implications.

As mentioned before, in the last decade the hs-cTn became an important tool in paediatric patients, not only for the identification of myocardial injury in several age-specific diseases but also in predicting chemotherapy-induced cardiotoxicity or the outcomes of other cardiac and noncardiovascular disease. But, as the biomarker is evaluated from blood samples, which may be difficult to obtain especially from newborns, in this population a new method of detection would simplify the evaluation and serial assessment which is often needed. Recently, hs-troponins have been detected in the urine of healthy subjects, with a significantly higher value in the urine of those with hypertension, suggesting the possibility of detecting hs-cTn in other biological fluids, by means less invasive than before [73]. Moreover, as salivary hs-cTnT levels correlated significantly with serum hs-TnT level in patients with AMI in a study conducted by Mirzaii et al. [74], further research is needed in order to develop new noninvasive determination methods, which could lower the risk of blood-borne diseases and allow nontrained personnel and even the patient to monitor this biomarker.

Another major challenge is represented by the variability of this biomarker and even if the ESC Guidelines do not recommend age-dependent cut-off points, as multiple studies above mentioned [63–65] showed, using adjusted cut-off values may not only improve the prognostic role but also the management of neoplastic children. Moreover, as the interpretation of the hs-cTn changes is still challenging in the patients with basal elevation such as CKD or critically ill patients, further studies are needed to assess the clinical implication of using specific cut-off values in these patients.

What is more, while there is solid evidence regarding the prognostic association of hs-cTn-T with CV morbidity and mortality, it is still unknown if the biomarker could be a treatment-effect modifier in randomized controlled trials. The recent EMPEROR-Reduced trial explored this gap in evidence and while the results demonstrated a cardioprotective benefit of empagliflozin in heart failure patients irrespective of baseline levels of hs-cTn-T, post-hoc analysis will probably also show if the biomarker modifies treatment responses with beneficial effects [75].

Nevertheless, as the COVID-19 pandemic still represents an important medical, economic and social issue, future research is needed to better understand it. Even if the prognostic role of hs-cTn in these patients have already been demonstrated and multiple studies suggest a better risk stratification when serial assessments are made, it became a major challenge to exclude an ACS in these patients, as many of them present not only an important hs-cTn elevation (frequently with dynamic changes) but also EKG and echocardiography modifications. In a study including 918 patients tested positive for COVID-19, 20.7% fulfilled strict criteria for myocardial injury, but only 2.1% suffered a classical ACS [70]. As an invasive diagnosis strategy, mainly coronary angiography is often needed to be able to exclude an ACS, these findings emphasis the urgent need for future research in order to better integrate the hs-cTn in the management of COVID-19 patients and to avoid unnecessary investigations.

## 5. Conclusions

The continuous improvement of cardiac biomarkers led to the development of the high-sensitivity assays of cardiac troponins I and T, which have high sensitivity and specificity. The use of the new generation hs-cTn is now recommended by the international guidelines as a centre piece in the diagnosis of myocardial infarction. Although highly cardiac-specific, this biomarker is not as high as ACS specific, as many cardiac or even noncardiac conditions may cause hs-cTn elevation. Even so, its use for the rapid rule-in/rule-out of ACS is safe and efficient, but keeping in mind that analytical performance of immunoassay methods and the demographic characteristics such as age or sex may affect the 99th percentile URL value, with further studies needed to validate if specific cut-offs could improve its performance. Moreover, the hs-cTn is a valuable prognostic tool not only for patients with acute coronary syndromes but also for a large spectrum of pathologies. Furthermore, hs-cTn is one of the most important biomarkers used to detect myocardial injury and predict outcomes in paediatric patients, and a possible future method of determination besides blood samples could augment its use in this population. Nevertheless, in the current COVID-19 era, the assessment of hs-cTn proved to be an important instrument for improving risk stratification, treatment personalization, and outcome prediction. As there are still many challenges to be faced regarding the daily use of hs-cTn in different clinical scenarios, more research and cross-disciplinary collaboration are necessary to improve their performance.

## Figures and Tables

**Table 1:** Sensitivity and sensibility of cardiac biomarkers.

		0 h	2-6 h	6-12 h	12-24 h
Myoglobin	Sensitivity (%)	59.5	84.0	75.0	59.3
		0 h	3-6 h	6-12 h	

CK-MB mass	Sensitivity (%)	57.1	89.8	97.0	
		0 h	2-6 h	6-12 h	12-24 h

Troponin T	Sensitivity (%) Cut-off 0.10 ng/l	60.2	79.4	99.2	97.7
	Sensitivity (%) Cut-off 0.04-0.05 ng/l	65.0	89.9	100	—
		0 h	2-6 h	6-12 h	12-24 h

Troponin I		44.4	68.8	100	88.9

CK-MB = creatine kinase isoenzyme MB.

**Table 2:** Analytical characteristics of high-sensitivity methods used to determine hs-cTn.

Analytical characteristic	Definition and description	Remarks
LoB	Lowest signal generated in a sample without troponin	Lower values are desirable

LoD	Value obtained in a sample with the lowest concentration of troponin	Lower values are desirable

LoQ	Lowest concentration of troponin that can be determined with acceptable repeatability and reproducibility (a <10% error)	Lower values are desirable

99th percentile (general)	Troponin concentration detected in 99% of truly healthy individuals	1% of truly healthy individuals can have false-positive results, for unknown reasons

99th percentile (sex-specific)	Troponin concentration detected in 99% of truly healthy individuals, taking into account the sex	In men, the 99th percentile upper limit is about 1.5-2 times higher than that in women

Cut-off value	Minimum troponin concentration for diagnosis of AMI	The level of 99th percentile is used as a reference

CV%	Random variation of measurements in the same sample	(i) <10% (preferred)—high precision (ii) 10-20% (acceptable)—nonhigh accuracy (iii) >20% (unacceptable)—inaccurate

Percentile values < 99th percentile in healthy subjects	Number of healthy individuals with detected troponin level in blood	Values range between LoD and the 99th percentile

99th percentile/LoD ratio		(i) <1 (acceptable)—highly sensitive (ii) >10—extremely sensitive (iii) >20—ultrasensitive

LoB = limit of blank; LoD = limit of detection; LoQ = limit of quantitation; CV% = coefficient of variation; AMI = acute myocardial infarction; hs-cTn = high-sensitivity cardiac troponins.

**Table 3:** Causes for hs-cTn elevation.

Myocardial injury related to acute myocardial ischaemia in ACS	Myocardial injury related to acute ischaemia resulting from oxygen supply/demand imbalance		Other causes for myocardial injury, without CAD		False-positive results
	Reduced coronary perfusion	Increased myocardial oxygen demand	Cardiac related	Multifactorial, systemic or indeterminate	
Plaque rupture followed by thrombosis	Coronary vasospasm	Sustained tachyarrhythmia	Heart failure	Sepsis, infectious disease	Cross-sectional reactions of anticardiac directed antibodies
	Coronary embolism	Severe hypertension	Cardiomyopathy	Pulmonary embolism or pulmonary hypertension	
	Microvascular dysfunction		Takotsubo syndrome	Chronic kidney disease	Heterophilic antibodies, rheumatoid factor, biotin, alkaline phosphatase
	Coronary artery dissection		Myocarditis pericarditis	Stroke	
	Sustained bradyarrhythmia		Coronary revascularization procedures	Subarachnoid haemorrhage	
	Hypotension/shock		Catheter ablation	Infiltrative diseases	Violation of the preanalytic stage: lipemia, ictericity, hemolysis, fibrin clots, or a malfunction of the analyzer
	Respiratory failure		Cardiac procedures	Critically ill patients	
	Severe anemia		Defibrillator shocks	Strenuous exercise	
			Cardiac contusion	Cardiotoxic agents (chemotherapy, narcotic drugs, adreno- and sympathomimetics)	
				COVID-19	

ACS = acute coronary syndrome; CAD = coronary artery disease.
